# *Erratum*: Vol. 65, No. 36

**DOI:** 10.15585/mmwr.mm6646a5

**Published:** 2017-11-24

In the report “Vital Signs: Disparities in Antihypertensive Medication Nonadherence Among Medicare Part D Beneficiaries — United States, 2014,” on page 973, in Table 3, the data for states beginning with “N” should have read as follows:

**Table 3:** Antihypertensive medication nonadherence among Medicare Part D beneficiaries aged ≥65 years, by state and territory, United States, 2014

State/Territory	No. beneficiaries	AHM fills				Annual AHM spending			
		Total (millions)	Mean maximum treatment intensity*	Percent fixed-dose combinations	Mean days’ supply per fill	Total spending per beneficiary ($)	Out-of-pocket spending per beneficiary ($)	Percent of out-of-pocket spending attributed to AHM	Percent nonadherent^†^
**Nebraska**	**108,367**	**1.49**	**2.20**	**8.7**	**46.4**	**302**	**111**	**17.9**	**22.6**
**Nevada**	**135,396**	**1.38**	**2.15**	**8.6**	**59.1**	**250**	**73**	**15.6**	**28.2**
**New Hampshire**	**66,971**	**0.71**	**2.10**	**5.2**	**60.4**	**285**	**99**	**18.6**	**20.5**
**New Jersey**	**532,767**	**5.49**	**2.22**	**11.2**	**60.5**	**472**	**117**	**21.5**	**25.3**
**New Mexico**	**103,182**	**1.06**	**2.08**	**7.0**	**56.9**	**261**	**77**	**17.9**	**29.8**
**New York**	**1,243,971**	**15.11**	**2.23**	**9.6**	**52.3**	**404**	**83**	**20.2**	**25.3**
**North Carolina**	**615,702**	**8.05**	**2.24**	**10.4**	**47.4**	**307**	**93**	**17.4**	**28.1**
**North Dakota**	**42,929**	**0.54**	**2.24**	**7.3**	**53.5**	**272**	**109**	**17.5**	**18.7**

**Abbreviation:** AHM = antihypertensive medication.

* Mean of the maximum number of AHM classes on hand at any one time per beneficiary; proxy for blood pressure treatment intensity.

^†^ Nonadherence is defined as patients not following their health care professional’s instructions concerning taking their prescribed medication. Using the proportion of days covered methodology, beneficiaries were considered nonadherent if they had access to AHM for <80% of the days from the date of their first AHM fill through the end of 2014 or their death in 2014.

